# Distribution patterns and environmental risk assessments of microplastics in the lake waters and sediments from eight typical wetland parks in Changsha city, China

**DOI:** 10.3389/fpubh.2024.1365906

**Published:** 2024-05-09

**Authors:** Junyi Yao, Jiang Li, Jialing Qi, Mengrui Wan, Liling Tang, Hui Han, Kai Tian, Shaobo Liu

**Affiliations:** ^1^The Department of Environmental Design, School of Architecture and Art, Central South University, Changsha, China; ^2^Henan Field Observation and Research Station of Headwork Wetland Ecosystem of the Central Route of South-to-North Water Diversion Project, School of Life Sciences and Agricultural Engineering, Nanyang Normal University, Nanyang, Henan, China

**Keywords:** microplastics, park, distribution characteristics, ecological risk, Raman spectrometer

## Abstract

The quality of water in urban parks is closely related to people’s daily lives, but the pollution caused by microplastics in park water and sediments has not been comprehensively studied. Therefore, eight typical parks in the urban area of Changsha, China, were selected, and Raman spectroscopy was used to explore the spatial distributions and compositions of the microplastics in the water and sediments, analyze their influencing factors, and evaluate their environmental risks. The results showed that the abundances of surface water microplastics in all parks ranged from 150 to 525 n L^−1^, and the abundances of sediment microplastics ranged from 120 to 585 n kg^−1^. The microplastics in the surface water included polyethylene terephthalate (PET), chlorinated polyethylene (CPE), and fluororubber (FLU), while those in the sediments included polyvinyl chloride (PVC), wp-acrylate copolymer (ACR), and CPE. Regression analyses revealed significant positive correlations between human activities and the abundances of microplastics in the parks. Among them, the correlations of population, industrial discharge and domestic wastewater discharge with the abundance of microplastics in park water were the strongest. However, the correlations of car flow and tourists with the abundance of microplastics in park water were the weakest. Based on the potential ecological risk indices (PERI) classification assessment method, the levels of microplastics in the waters and sediments of the eight parks were all within the II-level risk zone (53–8,549), among which the risk indices for Meixi Lake and Yudai Lake were within the IV risk zone (1,365–8,549), which may have been caused by the high population density near the park. This study provides new insights into the characteristics of microplastics in urban park water and sediment.

## 1 Introduction

Microplastics are small plastic particles with diameters of less than 5 mm and include polyethylene (PE), polypropylene (PP), polyvinyl chloride (PVC), polyethylene terephthalate (PET) and polystyrene (PS) (1). As plastic products have been widely used globally and in various aspects of human life (2), these common types of microplastics are heavily discovered in high-density human settlements and are often integrated in surface water. The most common types of microplastics in lake systems worldwide were PET, PVC, PP, and PE (3, 4). The most common polymer components of microplastics were: PP (25.0%), PET (22.6%) and PE (19.0%) in Asian lakes; PP (50.0%) and PS (27.3%) in European lakes, PP (33.8%) and PE (25.1%) in American lakes, and PVC (44.5%) and PET (40.9%) in African lakes. For lake sediment, the largest proportion of microplastics in Asia are PET (40.4%) and PE (20.1%), for Americas they are PE (67.7%) and PP (16.0%), for Africa they are PVC (61.9%) and PET (28.3%) (5, 6). Due to the widespread use of plastic products, human dumping, and slow degradation, micr. In addition, the microplastics are also transmitted through the food chain and even pose a threat to human health (7, 8). Plastic waste pollution in the environment is becoming increasingly severe (9, 10). On the one hand, microplastic waste enters land and water environments through random dumping, sludge reuse, air settling, and rainwater runoff and may be absorbed by soil plants, ingested by animals, or entangled with aquatic animals, leading to suffocation and death (11, 12). On the other hand, the plastics themselves contain various additives and toxic substances as well as adsorbed heavy metals and persistent organic pollutants, which are ingested by organisms and transferred to others, causing secondary damage to those organisms (13, 14).

There are two main sources of microplastics in the environment: (1) Primary microplastics, which are directly manufactured with micrometer sizes, such as industrial plastic raw materials that may leak into the environment during industrial production, transportation, and sales (15). The plastic particles in drugs or toiletries may be directly released into domestic sewage water without being treated by a water treatment system, or may escape from a sewage treatment plant and enter the environment due to incomplete removal (2). Secondary microplastics are formed by fragmentation and decomposition of large plastic wastes in the environment under physical, chemical, and biological conditions (16, 17). For example, ultraviolet radiation and high temperatures cause changes in the chemical substances in plastic products and increase their vulnerability to fragmentation into microplastics (18, 19). In recent years, freshwater microplastic pollution has been investigated for lakes in Europe, North America, and China (20, 21). The characteristics of microplastic composition in 39 urban and rural lakes in India were investigated and found that the average content of microplastics in water and sediment of urban lakes was 88.06 items L^−1^ and 115.24 items kg^−1^, respectively, while the average content of microplastics in rural lakes was 42.98 items L^−1^ and 53.29 items kg^−1^ (22). Moreover, research areas with high population density, large sewage discharge, and many residential areas and urban centers had higher microplastic abundance. Fibers are the dominant group and polyethylene and polypropylene are the most commonly found polymers. The abundance of microplastics in 41 urban and rural rivers was analyzed (23). The results showed that the content of microplastics in rural rivers was significantly lower than that in urban rivers (ranging from 0 to 230 pcs m^−3^), which was positively correlated with population density. During the transition from rural to urban areas, the content of microplastics increased sharply, which was consistent with the emergence of sewers. Trawls have been used to collect floating microplastics and determine the levels of microplastics in the lakes of several European countries, such as Switzerland. Among them, the highest abundance of microplastics was in Lake Geneva, Switzerland and reached 48,146 n km^−2^ (24). The average abundance of microplastics in the water of the Great Lakes basin in North America was 43,157 n km^−2^ (25). In addition, investigations were conducted on microplastics pollution in several major lakes in the middle and lower reaches of the Yangtze River in China (500–25,800 n m^−3^), lakes in Wuhan, China (1,660–8,925 n m^−3^), and lakes in central Italy (0.82–4.42 n m^−3^) through *in situ* screening or sampling and laboratory filtration methods (26–28). The spatial distributions of microplastics in different freshwater environments were compared, including urban streams (Shanghai), rivers (the Suzhou River and Huangpu River), estuaries (the Yangtze River estuary), and coastal waters (the East China Sea) (29). Their results revealed significant spatial differences in microplastic abundances, and the average concentrations of microplastics in freshwater environments were greater than those in estuaries and coastal waters. Overall, microplastic pollution in freshwater in China is severe, with a greater abundance than in other regions of the world. This may be due to the high population density and high plastic usage in China’s research areas (30). However, there is currently relatively little research on the abundance of microplastics in urban park water. Urban parks often adjoin residential areas, leading to the entry of large amounts of microplastics into the water and sediment. Therefore, exploring the abundance and ecological risks of microplastics in the waters and sediments of typical urban parks is crucial for ensuring peoples’ daily health.

With deepening of the research on microplastics, increasing attention has been given to collection and detection methods for microplastics (31–33). At present, there are three main methods for collecting microplastics in the field: (1) Direct picking methods, which are aimed at larger plastic particles (1–6 mm), such as primary raw materials that are easier to identify in the sediment or soil and can be directly picked (34). (2) The concentrated sample method involves immediately separating and preserving the microplastics in a sample at the sampling site. Microplastics in water are typically collected with trawl nets, while microplastics in sediments are collected with stainless steel screens (35). (3) The bulk sampling method involves the use of buckets or glass bottles to collect samples from the field and bring them back to the laboratory for analyses, targeting contents that are difficult to identify with the naked eye (36). Directly picked microplastics can be brought back to the laboratory for direct microscopic observation. The microplastics screened by trawl nets or stainless-steel sieves must be treated with a digestion solution to remove the biomass and then filtered for observation. After the sediment sample was dried, the microplastics were floated into the upper solution via the density separation method and then filtered and digested for microscopic observation. The extraction and separation of microplastics is an important step in laboratory microplastics analyses, in which both the digestion solution and suspension are crucial. The most commonly used digestion solutions currently available are 10% KOH and 30% H_2_O_2_ (37). At present, the mainstream methods for identifying microplastics include micro-infrared spectrometry, microRaman spectrometry, and scanning electron microscopy/energy spectrometry (38, 39). The size detection limit of the microRaman spectrometer was approximately 1 μm, which is widely used (32, 40).

Changsha city is located in the northeastern region of Hunan Province and is the political, economic, cultural, scientific, educational, and commercial center of Hunan Province. The type of industrial factory in Changsha city were engineering machinery, automobile and parts manufacturing, biomedicine, and food manufacturing. The main urban area of the city measures 1909.86 km^2^, with a permanent population of 10 million people. Most of the rivers in Changsha city belong to the Xiangjiang River system. There are more than 50 large parks in Changsha city. Therefore, eight typical parks were selected for surface water and sediment collection, and microRaman spectroscopy was used to study (1) the abundance and composition of the microplastics; (2) the factors influencing the levels of the microplastics; and (3) the ecological risk of the microplastics. This study was expected to identify and reveal the key factors affecting the degree of microplastic pollution in urban park water, providing a reference for a deeper understanding of the microplastic pollution situation in urban parks and also providing a reference for urban environmental health management.

## 2 Materials and methods

### 2.1 Research area

Changsha city is located in the northeastern region of Hunan Province, downstream of the Xiangjiang River and on the western edge of the Changliu Basin (111°53′ E and 27°51’ N). The length from east to west is approximately 230 km, and the width from north to south is approximately 88 km. Most of the rivers in Changsha city belong to the Xiangjiang River system. Moreover, 15 tributaries flow into the Xiangjiang River, mainly the Liuyang River, Laodao River, Jinjiang River, and Weishui River. There are 302 tributaries with lengths greater than 5 km, including 289 in the Xiangjiang River basin. The hydrological characteristics of Changsha include a complete water system and a dense river network. There is a large amount of water and abundant water energy resources. The average annual surface runoff within the city is 8.265 billion m^3^, with runoff depths ranging from 550 to 850 mm. The annual average runoff of the Xiangjiang River flowing through Changsha city is 69.25 billion m^3^, and the river is navigable throughout the year.

### 2.2 Sample collection

**Figure 1:**
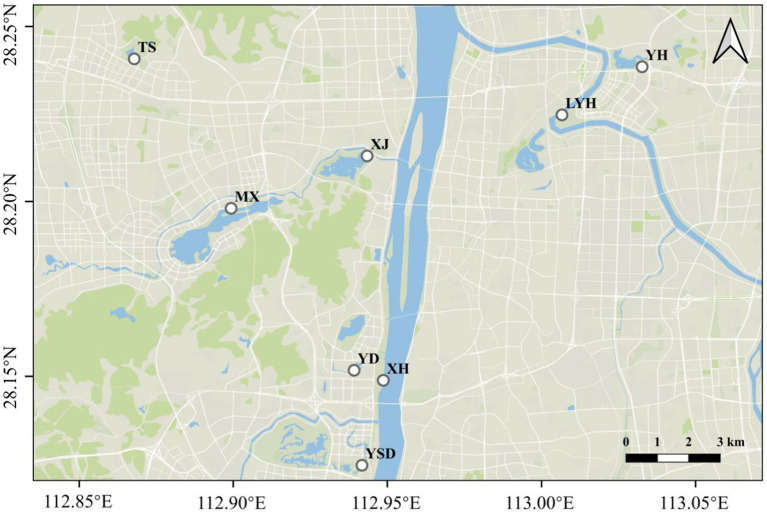
Schematic diagram of the sampling locations.

Eight typical wetland parks in Changsha city were selected as the research objects (Figure 1): Yudai Lake (YD), Liuyang River (LYH), Meixi Lake (MX), Jinshan Lake (TS), Xiangjiang Jiangtan Park (XH), Xianjia Lake (XJ), Yuehu Park (YH), and Yanghu Wetland Park (YSD). Two surface water samples and two sediment samples were collected from each park, and the sediment samples were collected from below the water sample. The samples were collected in March, 2023. All tools were cleaned with ultrapure water at least 3 times before sampling. A 2 L iron sampling bucket was placed at a depth of 20–30 cm underwater for sampling. Fifteen liters of water was collected at each sampling point and filtered through a 0.045 mm stainless steel sieve. Then, all of the solids retained on the sieve were rinsed with deionized water into a sampling bottle for preservation. This study included microplastics with sizes within the range 0.045–5 mm. The sampling points for sediment microplastic collection were consistent with the collection times and locations of the microplastics in the surface water. The collections of surface sediments from the lakes were carried out with a Peterson grab at the site location (41). Five hundred grams of sediment was packed into a self-sealing bag, labeled, and brought back to the laboratory for refrigeration.

### 2.3 Extraction of microplastics

Each water sample was added to 20 mL of 30% H_2_O_2_ solution in the laboratory and digested at room temperature for 24 h until visible organisms were removed from the samples. A total of 800 mL of a saturated NaCl solution (1.2 g cm^−3^) was added to the reaction mixture, which was thoroughly stirred and allowed to stand for 24 h before extracting the supernatant. Glass ultrafine fiber filter paper (0.45 μm) was used for vacuum filtration of the supernatant. Finally, the filter paper was stored in a culture dish and immediately covered with aluminum foil for future observation. Each sediment sample was placed separately in a glass culture dish and covered with aluminum foil to prevent indoor pollution. Then, the glass culture dish was placed in a drying oven and allowed to stand for 24 h at 60°C. After the sediment sample was completely dried, it was mixed evenly and initially screened through a 5 mm stainless steel sieve. Large impurities such as stones and branches were removed. Subsequently, 100 g of the dried sample was removed and placed in a 1 L beaker. A saturated sodium chloride solution was added to the beaker, which was subsequently stirred with a glass rod for 2 min and allowed to stand for 24 h. Finally, the supernatant was collected. This experimental process was repeated three times. All of the supernatants were collected and passed through a 0.45 μm nylon membrane filter (with a negative pressure of no more than 40 kPa). All of the substances on the filter membrane were transferred to a 200 mL beaker, and an excess of 30% H_2_O_2_ solution was added (37). The solution was then shaken at 65°C and 80 rpm for 24 h for digestion. The dissolved solution was again filtered through a nylon membrane with a pore size of 0.45 μm and a diameter of 47 mm. Finally, the membrane was placed in a clean glass Petri dish for inspection. The PM_2.5_ in the laboratory atmospheric was 8 μg m^3^. It is noteworthy that the extraction experiment of microplastics was conducted in a super clean workbench, where the air was filtered. In addition, in order to eliminate experimental errors caused by containers and air, we also set up a CK control, which only added digestion solution without adding water or sediment samples. The results of microplastic content in all samples were obtained after removing the plastic content in the CK control.

### 2.4 Identification of microplastics

The chemical compositions of the microplastics in the sample were identified with a Raman spectrometer and a laser infrared imaging spectrometer (Agilent 8,700 LDIR) (32). Briefly, the obtained filter membrane was immersed in an ethanol solution for ultrasonic treatment, so that the substances on the filter membrane were dispersed in the ethanol solution. The filter membrane in the ethanol solution was removed and cleaned multiple times with ethanol. Then, the ethanol solution was concentrated and added to a high reflection glass. After the ethanol was completely evaporated, LDIR testing was performed. The particle analysis method and microplastics spectrum library were used for the analyses. The automatic testing method was also used (matching degree > 0.65, particle size range 20–500 μm).

### 2.5 Quality control

To avoid contamination during extraction of the microplastics, glass containers were used during the experiments. All open containers were covered with aluminum foil to prevent microplastics from contaminating the experimental samples in the environment. The high-purity water used for cleaning the instruments and preparing the solutions was obtained via filtration through a 0.45 μm glass fiber membrane. Pure water filtered through a 0.45 μm glass fiber filter membrane was used for the blank experiment. The whole experimental process was consistent with the experimental steps for the above water samples. A total of 10 microplastic particles were found from the 3 blank samples, and the blank value was deducted from the following results.

### 2.6 Collection of environmental factor data

Environmental factors was collected that could affect the abundance of microplastics within a 3-kilometer radius of each park, mainly including the number of restaurants, express delivery, and supermarkets, population size, vehicle flow, the amount of household waste generated, industrial and domestic wastewater emissions and park visitors. The main methods of collection were door-to-door statistics, statistical yearbooks, and social development statistical bulletins.

### 2.7 Calculations of the ecological risks from microplastics

To evaluate the ecological risk of microplastic pollution in typical wetland parks, the potential ecological risk index (PERI) was calculated based on the abundance of microplastics and polymer types. The formula was:

$$\left\{ \begin{array}{l} Ek=TK\times \left(\frac{\mathrm{Ci}}{CO}\right)\\ Tk=Pk\times Sk\\ \mathrm{PERI}=\overset{M}{\underset{k=1}{\sum }}Ek \end{array}\right.$$

where *C*_i_ is the abundance of microplastics; *C*_0_ is the standard reference value, and Everaert, Van Cauwenberghe, De Rijcke, Koelmans, Mees, Vandegehuchte and Janssen (42) calculated the safe abundances of microplastics in surface water (6,650 n L^−1^) and sediment (540 n kg^−1^); *T*_k_ is the chemical toxicity coefficient of polymer *k*; *P*_k_ is the percentage of polymer *k*; *S*_k_ is the hazard fraction of polymer *k* (43), and the specific values are shown in Table 1; and *E*_k_ and PERI are the potential ecological risk factors and potential ecological risk indices, respectively. The threshold values for the potential ecological risk classification standards were I (PER<10), II (10 ≤ PER<100), III (100 ≤ PER<1,000), and IV (1,000 ≤ PER<10,000).

**Table 1:** Polymer hazard fraction.

Microplastics	*S*_k_	Microplastics	*S*_k_
Acrylate copolymer (ACR)	12	Polybutadiene (PD)	11
Polyethylene terephthalate (PET)	4	Polycarbonate (PC)	1,177
Polysulfone (JF)	11	Chlorinated polyisoprene (CP)	1
Polyurethane (PU)	7,384	Polymethyl methacrylate (PMMA)	1,021
Polyvinyl chloride (PVC)	10,551	Polypropylene (PP)	1
Polybutadiene (BR)	1	Ethylene-acrylic acid (EAA)	44
chlorinated polyethylene (CPE)	1	Polylactic acid (PLA)	47
Fluororubber (FLU)	1	Polyethylene (PE)	11
Phenolic resin (PR)	1,500	Polystyrene (PS)	30
Styrene butadiene styrene (SBS)	11	Polyvinyl butyral (PVB)	6
Ethylene vinyl acetate (EVA)	9	Methyl methacrylate styrene (MBS)	2,788

### 2.8 Data analysis

The abundance of microplastics in the water and sediment of the parks was expressed in n L^−1^ (the number of microplastics contained in each liter of water sample) or n kg^−1^ (the number of microplastics contained in each kilogram of sediment sample). The data were analyzed with R version 4.2.2.^1^ Heatmaps were generated to demonstrate the spatial distributions of the microplastics across the sampling sites. To better understand the differences among the various types of microplastics and city parks, the abundance data were standardized with *Z* score transformation, and bidirectional clustering was conducted on both the microplastic types and sampling sites based on the Bray–Curtis distance. Spearman correlation analyses were used to evaluate the relationship between the microplastic abundance in surface water and that in sediment.

## 3 Results

### 3.1 Spatial variations of microplastics in typical wetland parks

**Figure 2:**
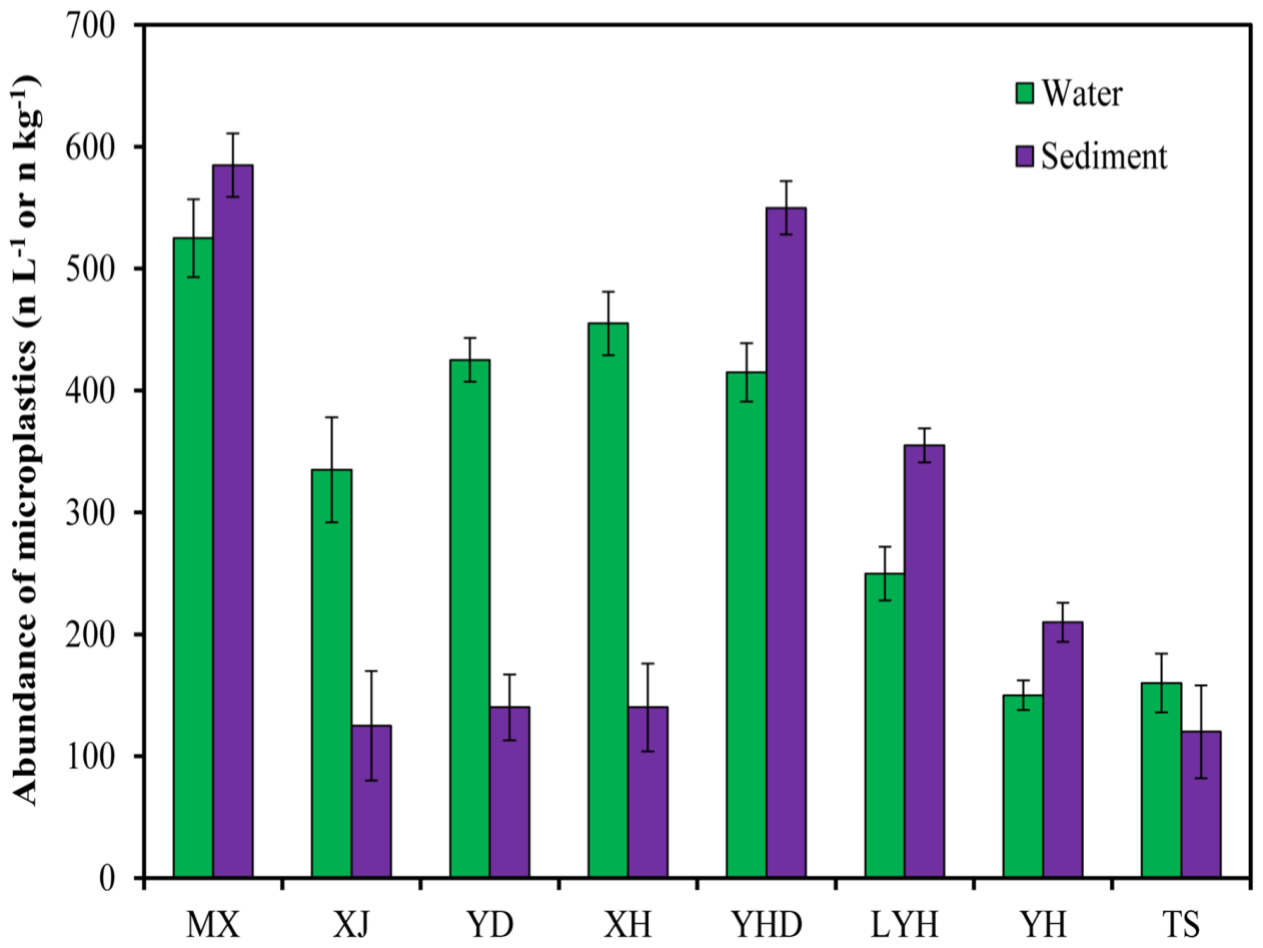
Spatial variations trend of microplastics abundance in typical wetland parks.

The microplastic abundances in the surface waters and sediments of eight typical parks in Changsha are shown in Figure 2. The abundances of microplastics in the surface waters of all parks ranged from 150 to 525 n/L, and the abundances of microplastics in the sediments ranged from 120 to 585 n/kg. Among the microplastics in the surface water, MX had the highest abundance (525 n L^−1^). This may be because MX is located in the center of Changsha, surrounded by residential areas around the lake, and with more artificial labor. The abundances of microplastics in XH, YD, and YHD were also relatively high, at approximately 400 n L^−1^. This may be due to the high pedestrian flow in the parks. For example, YD is located on the campus of Central South University, and YHD is the largest ecological wetlands park with a high number of tourists. The abundances of microplastics in TS and YH were relatively low, approximately 150 n L^−1^. This may be related to their locations in the suburbs and the low numbers of tourists. Among the abundant microplastics in the sediments, MX and YHD also had the highest levels of microplastics (585 n kg^−1^), which was consistent with the levels of microplastics in the surface waters, confirming that MX had the most severe microplastic pollution. Surprisingly, the abundances of microplastics in the sediments of TS, XH, XJ, YD, and YH were relatively low, at approximately 120 n kg ^−1^, which was inconsistent with the microplastic levels in the surface water. These results indicated that the abundance of microplastics in surface water does not represent the abundance of microplastics in the sediment. It was also possible that some microplastics were distributed in water, while others were distributed in sediment.

### 3.2 Composition of microplastics in typical wetland parks in Changsha

**Figure 3:**
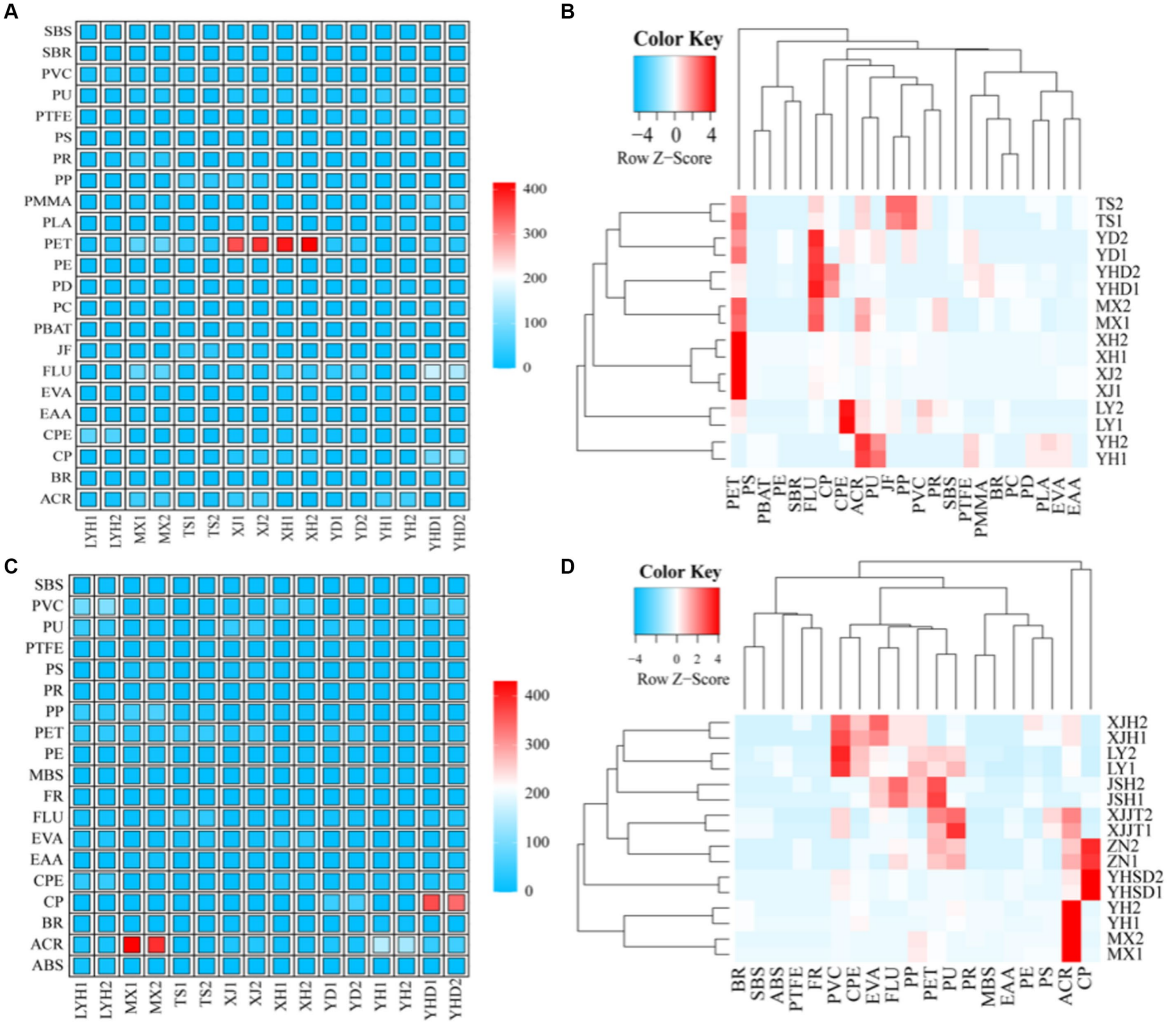
Compositions of the microplastics in typical wetland parks. **(A)** Composition of microplastics in the surface water; **(B)** cluster analysis of the microplastics in the surface water; **(C)** composition of the microplastics in the sediment; and **(D)** cluster analysis of microplastics in the sediment.

We arranged the samples according to their geographical information, such as MX and XJ (located in the middle, close to each other, with the same direct water source); YD, XH, and YSD (located in the south, all close to the Xiangjiang River); LYH and YH (located in the northeast, close to each other); TS (located in the northwest). The distribution patterns of various microplastics species among the different sampling points are shown in Figure 3. A total of 23 different polymers were identified by separating the microplastics from the surface waters of the eight parks (Figure 3A). Among them, a total of 10 polymers were identified in the surface water of LYH, and CPE showed the highest abundance at 80 n L^−1^. A total of 15 polymers were identified in the surface water of MX, and PET and FLU showed the highest abundances at 85 and 95 n L^−1^, respectively. A total of 10 polymers were identified in the surface water of TS, and PET, JF, and PP showed the highest abundances at 35, 30, and 35 n L^−1^, respectively. A total of 16 polymers were identified in the surface water of XH, and the highest abundance was that of PET at 305 n L^−1^. A total of 12 polymers were identified in the surface water of XJ, and the highest abundance was that of PET occurring at 395 n L^−1^. A total of 10 polymers were identified in the surface water of YD, and the highest abundances were those of PET and FLU at 30 and 40 n L^−1^, respectively. A total of 11 polymers were identified in the surface water of YH, and the highest abundances were those of ACR and PU at 45 and 35 n L^−1^, respectively. A total of 16 polymers were identified in the surface water of YHD, and the highest abundance was that of FLU at 165 n L^−1^. Based on the Bray–Curtis distance, a hierarchical clustering analysis of the microplastic compositions in the water was carried out (Figure 3B). The results showed that PET, unlike the other microplastics, exhibited a larger proportion in the waters from all sites except YH. The other microplastics were divided into three categories. The first category included PS, PBAT, PE, and SBR, which were the smallest groups, especially in MX, YHD, YD, and TS. The second category included FLU, CP, CPE, ACR, PU, JF, PP, PVC, and PR, which exhibited substantial proportions in MX, YHD, YD, and TS and showed a complementary trend with LYH and YH (increasing and decreasing), while in XH and XJ, they were all among the middle group. The third category included SBS, PTFE, PMMA, BR, PC, PD, PLA, EVA, and EAA, which exhibited lower proportions in XH and XJ, while in MX, YHD, YD, JS, and YH, they were generally present at low levels but had greater levels in individual areas (Figure 3B).

A total of 19 different polymers were identified by separating the microplastics from the sediments of the 8 parks (Figure 3C). Among them, a total of 15 polymers were identified in the sediment of LYH, and PVC showed the highest abundance at 95 n L^−1^. A total of 11 polymers were identified in the sediment of MX, and the abundance of ACR was the highest at 430 n L^−1^. A total of eight polymers were identified in the sediment of TS, and PET and FLU showed the highest abundances at 40 and 30 n L^−1^, respectively. A total of 10 polymers were identified in the sediment of XH, and PU showed the highest abundance at 45 n L^−1^. A total of nine polymers were identified in the XJ sediment, and PVC and EVA showed the highest abundances at 35 and 25 n L^−1^, respectively. A total of 10 polymers were identified in the YD sediment, and CP, PU and ACR showed the highest abundances at 50, 25, and 24 n L^−1^, respectively. A total of nine polymers were identified in the YH sediment, and the highest abundance was that of ACR at 155 n L^−1^. A total of 13 polymers were identified in the YHD sediment, and the highest abundance was that of CP at 365 n L^−1^. Based on the Bray–Curtis distance, a hierarchical clustering analysis of the microplastic composition structure in the water was carried out (Figure 3D). In the sediments, ACR and CP had the highest abundances, and their distributions differed from those of the other microplastics. These were the main microplastic components in MX, YH, YHD, and YD. The other microplastics were divided into three major categories, and BR, SBS, ABS, PTFE, and FR were the least common, especially in YD, XH, TS, LYH, and XJ. The second category included PVC, CPE, EVA, FLU, PP, PET, and PU. In contrast to those in the first category, these were generally the most common components in YD, XH, JS, LYH, and XJ, but they were relatively infrequent in MX, YH, and YHD. The third category included PRs, MBSs, EAAs, PEs, and PSs, with distribution characteristics similar to those of the first category (Figure 3D).

### 3.3 Identification and analyses of typical microplastics

**Figure 4:**
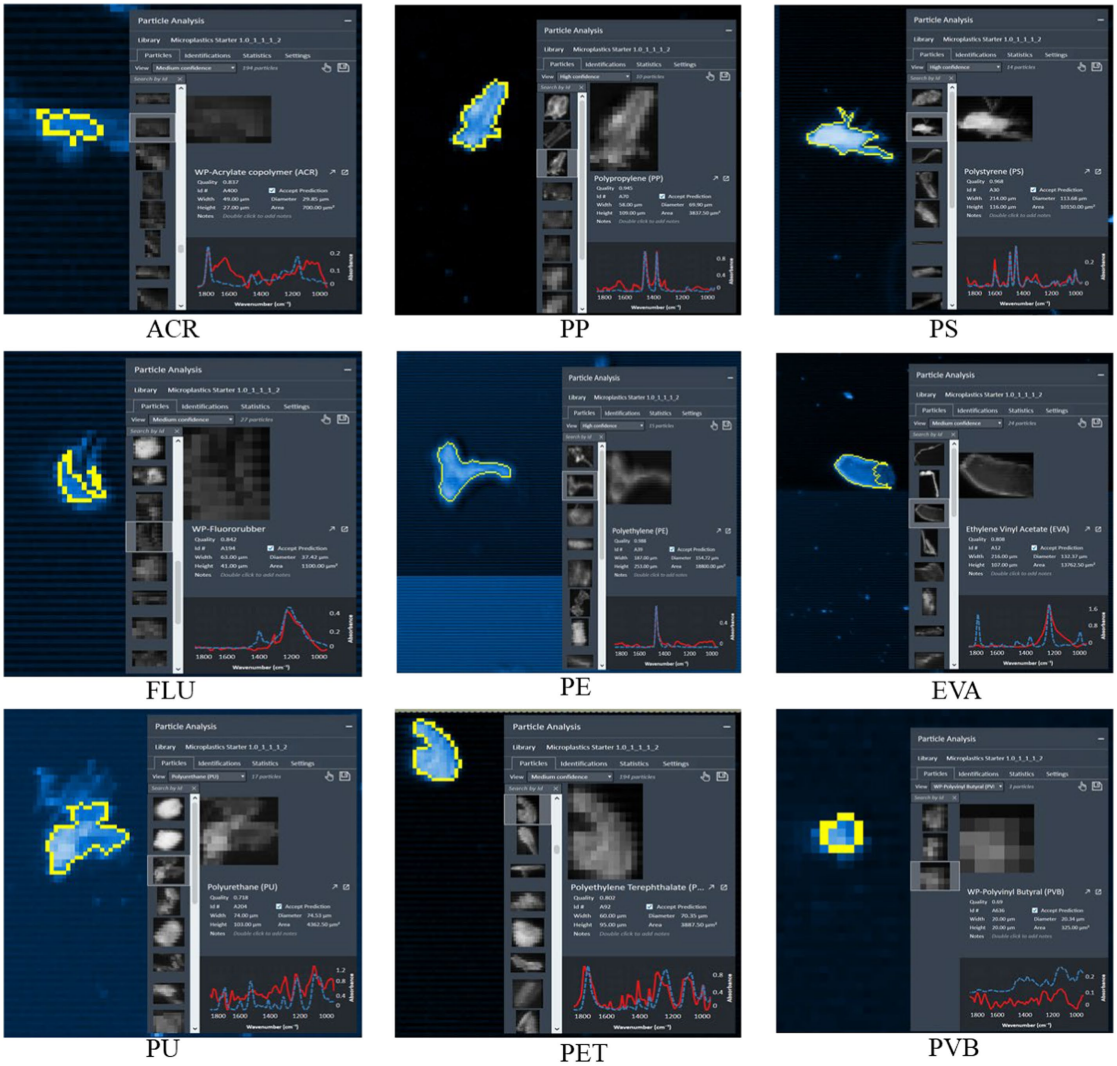
Typical microplastic identification diagram.

Acrylate copolymers (ACRs) generally refer to acrylic acid hydroxypropyl acrylate copolymers. Acrylate is a general term for esters of acrylic acid and its homologs, which are monomers used in manufacturing adhesives, synthetic resins, special rubbers, and plastics. Acrylate copolymers are used in the coatings, adhesives, leather, chemical fibers, papermaking, and printing industries. Raman spectroscopy revealed that ACR exhibited peaks at 1,445 and 1,716 cm^−1^ for a symmetric stretching vibration of N=C=O and an asymmetric stretching vibration for carbon atoms in the benzene ring (Figure 4). Polypropylene (PP) is formed by addition polymerization of propylene. PPs are widely used in the production of fiber products such as clothing and blankets, medical equipment, automobiles, bicycles, parts, transportation pipelines, chemical containers and food and drug packaging. The largest areas of polypropylene use in China include products such as woven bags, packaging bags, and tying ropes, which account for approximately 30% of the total consumption. Raman spectroscopy revealed that PP exhibited peaks at 1,458 and 1,363 cm^−1^, corresponding to a CH_2_ bending vibration and a CH_2_ rocking vibration, respectively (Figure 4). Polystyrene (PS) is synthesized by free radical addition of styrene monomers. PS is mainly used for insulation, shock resistance, packaging materials, and floating products. The universal type (R) is suitable for packaging materials, while the flame-retardant type (F) is suitable for building and insulation materials. PS can also be used to make foamed plastic products and films. Raman spectroscopy revealed that PS exhibited peaks at 1,000, 1,445, 1,530, and 1,602 cm^−1^, corresponding to the symmetric stretching vibrations of C-C bonds in the benzene ring, the symmetric stretching vibration of N=C=O, the stretching motion of C-C, and the stretching vibrations of C and O atoms in carboxyl groups, respectively (Figure 4). Fluorine rubber (FLU) provides heat resistance, oxidation resistance, oil resistance, corrosion resistance, and atmospheric aging resistance. It is used for mechanical seals, pumps, reactors, mixers, compressor casings, valves, various instruments, and other equipment, such as valve seats, valve stem fillers, diaphragms, and gaskets. It has been widely used in the aerospace, aviation, automotive, petroleum, and household appliance fields. Raman spectroscopy revealed that FLU exhibited peaks at 1,151 and 1,168 cm^−1^ for a C-C stretching vibration, a CH bending vibration, a CH_3_ oscillation, and a C-C rocking vibration, respectively (Figure 4). Polyethylene (PE) is an important fiber material and a common component of fragmented microplastics. PEs are often used in cling films, vest-style plastic bags, plastic food bags, milk bottles, buckets, water bottles, etc. PEs are mainly divided into three categories: linear low-density polyethylene (LLDPE), low-density polyethylene (LDPE), and high-density polyethylene (HDPE). Raman spectroscopy revealed that PE exhibited peaks at 1,439 and 1,461 cm^−1^, indicating cross-and swaying vibrations of CH_2_ groups (Figure 4). The ethylene-vinyl acetate copolymer is used as a hot melt adhesive (EVA) and is composed of the basic resin, tackifiers, viscosity regulators, and antioxidants. EVAs with vinyl acetate contents less than 5% are used produce films, wires and cables, LDPE modifiers, adhesives, etc. EVA products with vinyl acetate contents ranging from 5 to 10% are elastic films, etc. EVAs with vinyl acetate contents ranging from 20 to 28% are mainly used for hot melt adhesives and coating products. Raman spectroscopy revealed that EVA exhibited a peak at 1,220 cm^−1^ corresponding to a torsional vibration of CH_2_, a rocking vibration of CH, and a stretching vibration of C-C. Polyurethane (PU) can be made into foam plastics, elastomers, fiber plastics, fibers, leather shoe resins, coatings, adhesives and sealants, among which foam plastics account for the largest proportion. In addition, polyurethane, an important waterproof coating, is widely used in building roofs, exterior walls, roof slabs, basements, kitchens and bathrooms, roads and bridges, and other parts. Raman spectroscopy revealed that PU exhibited peaks at 1,283, 1,445, and 1,732 cm^−1^, corresponding to C-C stretching vibrations of benzene rings and carboxyl groups, a symmetric stretching vibration for N=C=O, a bending vibration of CH_2_, and a stretching vibration of C=O, respectively. Polyethylene terephthalate (PET) is an important synthetic fiber that can be spun and reprocessed into polyester. Polyesters are characterized by high strengths, good elasticities, strong heat resistances, and strong thermoplastic properties and have broad application prospects in the clothing, construction, and automobile industries. Raman spectroscopy revealed that PET exhibited peaks at 1,168, 1,435, and 1716 cm^−1^, corresponding to the stretching and rocking vibrations of C-C, the bending vibrations of CH_2_, and the asymmetric stretching vibrations of carbon–carbon bonds in the benzene ring, respectively. Polyvinyl butyral (PVB) is a condensate of polyvinyl alcohol and butyraldehyde and is mainly used in manufacturing laminated glass, coatings, and adhesives. In addition, PVB is widely used for bonding wood, ceramics, metals, plastics, leather, laminated materials, etc. Raman spectroscopy revealed that PET exhibited peaks at 1,283, 1,439, 1,445, and 1,461 cm^−1^ for the C-C stretching vibrations of the benzene rings and carboxyl groups, the symmetric stretching vibration of N=C=O, and the cross and sway vibrations of CH_2_.

### 3.4 Physical and chemical properties of the waters and sediments

**Figure 5:**
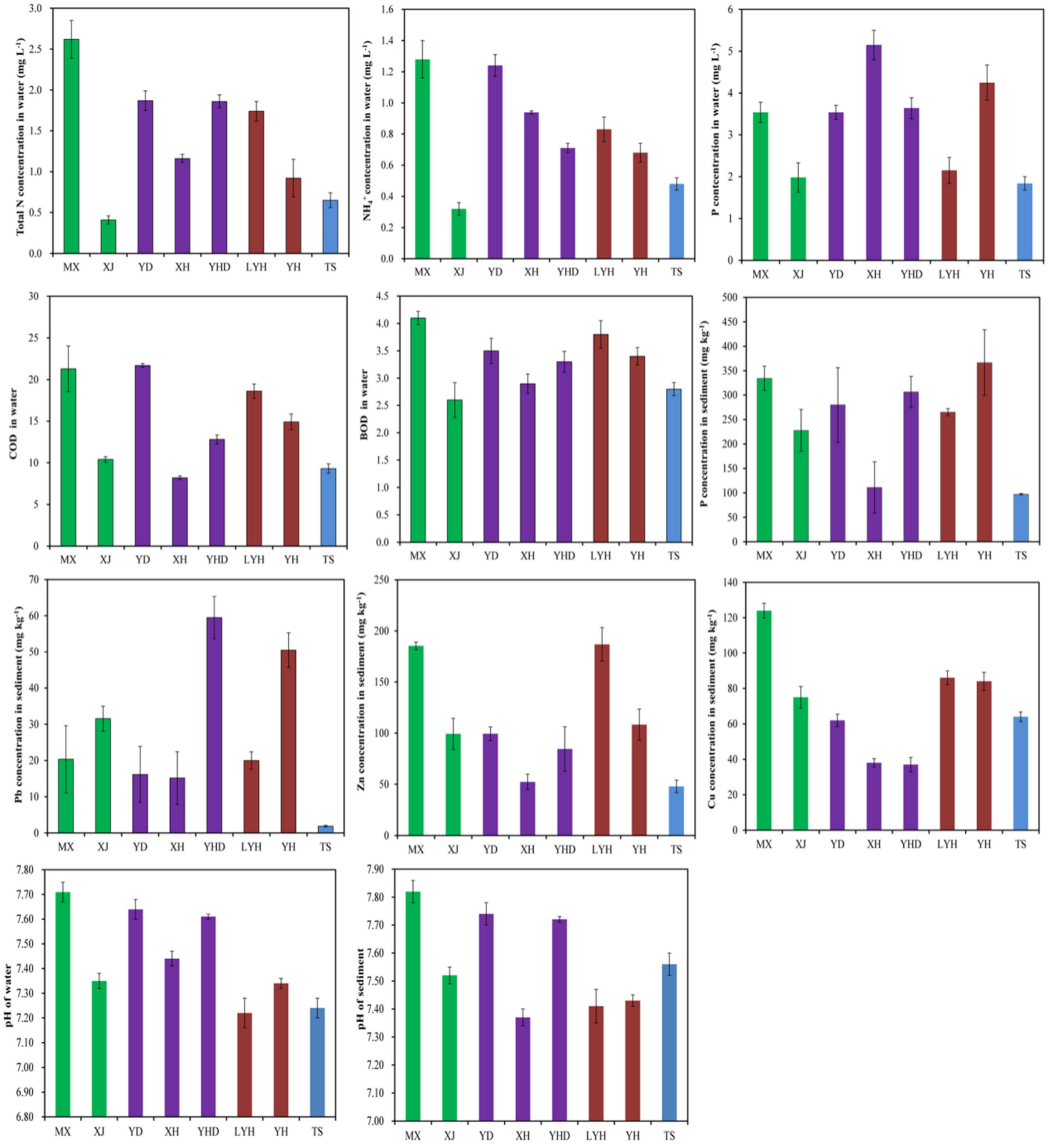
Physical and chemical properties of the waters and sediments in typical wetland parks in Changsha.

The physical and chemical properties of the surface waters and sediments in the eight parks were measured, and the results are shown in Figure 5. The total N content in the MX surface water was the highest at 1.28 mg L^−1^, while the total N contents in the TS and XJ waters were the lowest at 0.65 and 0.41 mg L^−1^, respectively. This was mainly because there were more residential areas and a greater population around MX, while there were no residential areas around TS and XJ, resulting in a more beautiful ecological environment. The NH_4_^+^ concentrations in the surface waters of MX and YD were 1.28 and 1.24 mg L^−1^, respectively, which were the highest among those in all the parks. The NH_4_^+^ contents in the TS and XJ water samples were the lowest, at 0.48 and 0.32 mg L^−1^, respectively. The total P content in the surface water of XH was 5.15 mg L^−1^, which was the highest among all parks. The total P contents in the surface waters of LYH, TS, and XJ were relatively low, with values of 2.15, 1.84, and 1.98 mg L^−1^, respectively. The pH of the park’s water samples range from 7.22 to 7.71, and the pH of the sediment samples range from 7.37 to 7.82, both of which were alkaline. Among them, the pH of MX water samples and sediment samples were 7.71 and 7.82, respectively, while the pH of YD water samples and sediment samples were 7.63 and 7.64, respectively. This may be due to the large number of residents around the park, which produced more alkaline water in daily life, leading to an increase in pH in the park’s water. The COD contents in the surface waters of MX and YD were 21.3 and 21.7, respectively, which were the highest among all parks. The COD contents in the TS and XH water samples were the lowest, at 9.3 and 8.2, respectively. The BOD contents in the surface waters of LYH and MX were 3.8 and 4.1, respectively, which were the highest among all the parks. The COD contents in the JS and XJ water samples were the lowest, at 2.8 and 2.6, respectively. Among the sediments of the eight parks, MX and YH had the highest total P contents at 334 and 366 mg kg^−1^, respectively. The total P contents in the TS and XH sediments were the lowest, at 97 and 110 mg kg^−1^, respectively. Based on measurements of the heavy metal contents in the sediments, the YH and YHD sediments had the highest Pb contents, at 50 and 59 mg kg^−1^, respectively. The Pb content in the TS sediment was the lowest, at 1.8 mg kg^−1^. The Zn contents in the LYH and MX sediments were the highest, at 198 and 182 mg kg^−1^, respectively. The Zn contents in the TS and XH sediments were the lowest, at 43 and 47 mg kg^−1^, respectively. The highest Cu content in the MX sediment was 124 mg kg^−1^. The Cu contents in the XH and YHD sediments were the lowest, at 38 and 37 mg kg^−1^, respectively.

### 3.5 Factors influencing the occurrence of microplastics

**Figure 6:**
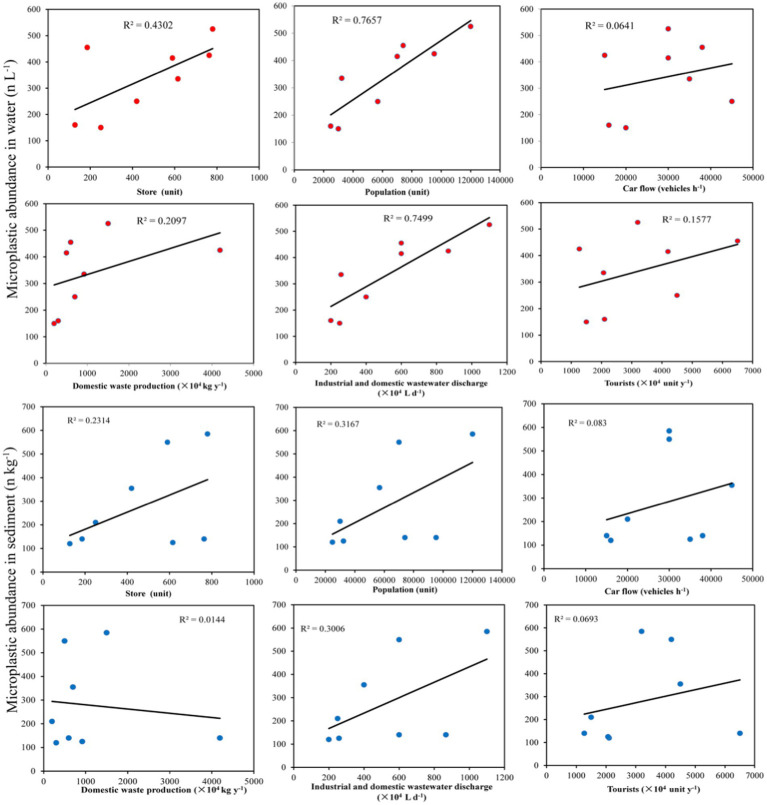
Relationships of the abundances of microplastics in water and sediment with human activities.

Human activity is the only source of microplastics and directly determines their distributions in the environment. A regression analysis revealed a significant positive correlation between human activities and the abundance of microplastics in park water (Figure 6). Among them, the correlations of population, industrial discharge and domestic wastewater discharge with the abundance of microplastics in park water were the strongest, followed by the correlations of the number of stores (supermarkets, express delivery stores, hospitals, and restaurants) and household waste production with the abundance of microplastics in park water. The correlations of car flow and tourists and with the abundance of microplastics in park water were the weakest. These results indicated that peoples’ daily family lives was the main factor affecting the distributions of microplastics in park water. Daily life generated various types of household waste and wastewater, which were discharged into rivers or bottom waters, thereby affecting the abundance of microplastics in park water. There were certain correlations between the abundance of microplastics in park sediments and the population, industrial and domestic wastewater discharge, and number of stores (supermarkets, express delivery stores, hospitals, and restaurants), while the correlations of the abundance of microplastics in park sediments with the amount of domestic waste generated, car traffic, and tourist traffic were relatively weak. This indicated that peoples’ daily family lives affected the abundances of microplastics in park sediments. However, the abundance of microorganisms in the sediment could have an impact on the abundance of microplastics. Therefore, it was necessary to continue exploring the environmental factors that affected the abundances of microplastics in sediments.

### 3.6 Ecological risk assessment of microplastics in typical wetland parks in Changsha

**Figure 7:**
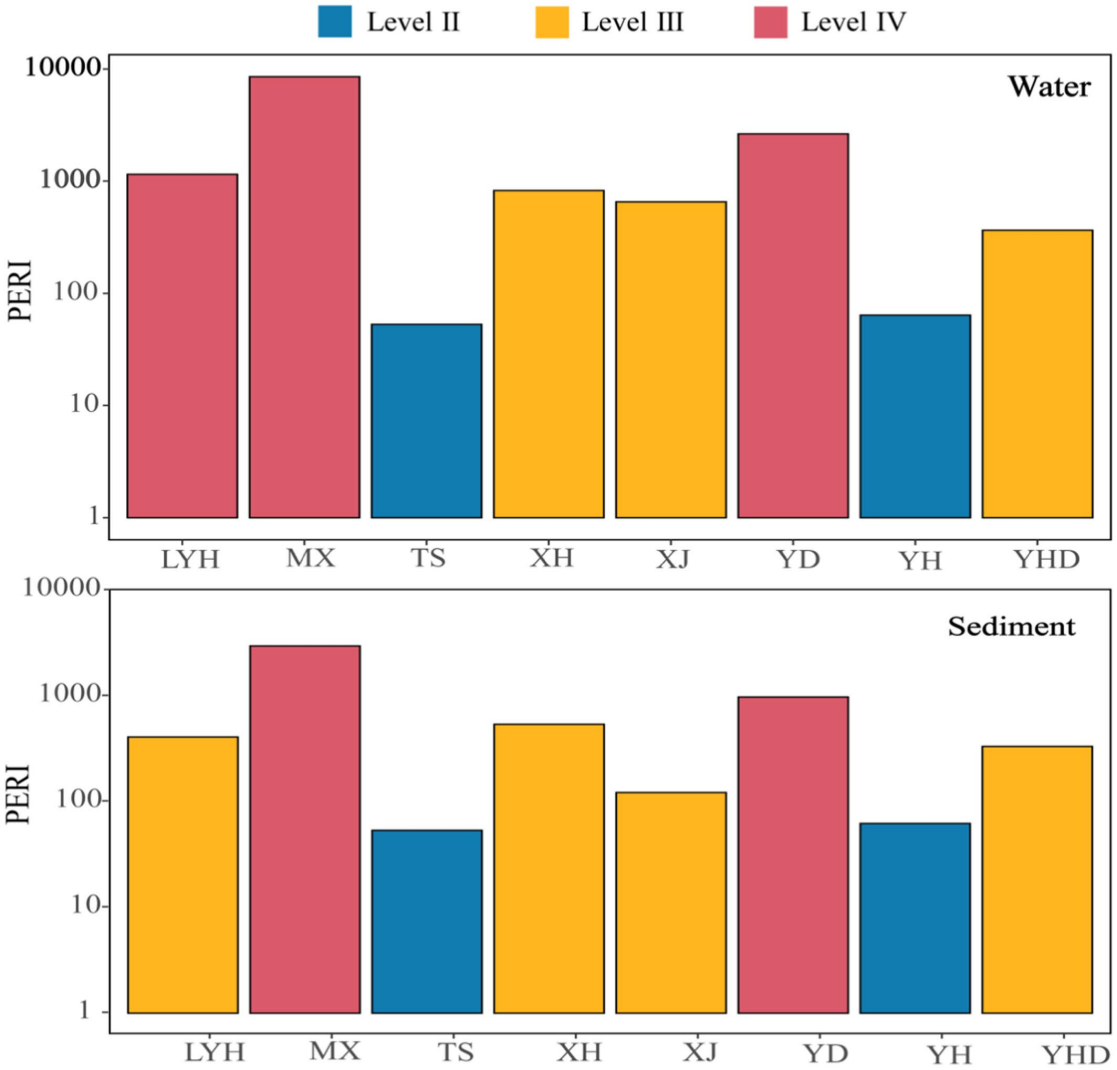
Ecological assessment of microplastics risks in typical wetland parks.

The potential ecological risk index (PERI) was used to evaluate the ecological risks caused by microplastic pollutants in the waters and sediments of the eight parks in Changsha (Figure 7). The PERIs of the microplastics in the various samples varied greatly, ranging from 53 to 8,549. Among the water samples, those from LYH, MX, and YD had PERI values in the IV risk zone; XH, XJ, and YHD had PERI values in the III risk zone; and TS and YH had PERI values in the II risk zone. In the sediment samples, MX and YD had PERIs in the IV risk zone; LYH, XH, XJ, and YHD had PERIs in the III risk zone; and TS and YH had PERIs in the II risk zone. These results indicated that the ecological risks from microplastics in the surface waters of the parks were basically consistent with those of the microplastics in the sediments. In addition, all the samples from the parks were above the Level II risk zone, indicating that there were ecological risks to the environments of these parks. The governance of microplastics and the management of the parks should be strengthened in the future.

## 4 Discussion

Microplastics come from various aspects of human production and life, mainly including discharge of wastewater containing textile microfibers (44), fragmentation of agricultural plastic films (45), wear and tear of tires and road markings (46) and decomposition of plastic waste (47), which together result in the widespread presence of microplastics in global water environments. Although some scholars have reported that some functional microorganisms could degrade microplastics (48, 49), this process is relatively slow, and microplastics are still easily ingested by aquatic organisms and spread through the food chain, which can affect the normal growth and development of organisms (8, 50). This article described the types and quantities of microplastics found in the waters and sediments of 8 typical parks in Changsha. The results showed that the abundances of surface water microplastics in all parks ranged from 150 to 525 n L^−1^, and the abundance of sediment microplastics ranged from 120 to 585 n kg^−1^. The uneven distribution of microplastics in the water environments may have been caused by many factors, such as the properties of the plastics, the hydrological conditions, the surrounding environment, and meteorological conditions (51, 52). It is generally believed that investigating larger water areas better reflects the levels of microplastics pollution in water. In this study, two sampling points were set for each study area, thereby increasing the representativeness of the samples. In addition, a stainless-steel screen with hole diameters of 0.45 mm was used for filtration during the sampling process, which made it possible to detect microplastics with small particle sizes. Small plastic particles constitute an important component of all microplastics. These microplastics with small particles were easily overlooked by samplers such as trawls with larger apertures. Small water samples (20 L) could also lead to differences in the microplastics concentrations of different sampling areas (53). In this study, parks with microplastics abundances greater than 400 n L^−1^ were distributed in the central urban area of Changsha. Among them, the highest abundance of microplastics was detected in MX, reaching 525 n L^−1^, followed by YD and XH, with 425 and 455 n L^−1^, respectively. There are many residents around these parks, so the degradation of plastic wastes in residential households may be the main source of microplastics in these lakes. In addition, due to the small surface areas of the lakes, the concentrations of floating particles in lakes may also lead to a high density for the microplastics suspended on the lake surfaces (54). The abundances of microplastics in TS and YH were not high, which may be attributable to the lower population densities around these sites. Since YHD is an important wetland tourist attraction in Changsha, the abundance of microplastics in the YHD water was as high as 415 n/L. The degradation of plastic wastes discarded by tourists and nearby residents may be one of the sources of microplastics in water (55).

Based on the findings from studies done with larger water bodies, the abundances of microplastics in these smaller water bodies were not very different from those in the large water bodies (56, 57). The major lakes and rivers in the middle and lower reaches of the Yangtze River in China were investigated and found that the abundances of microplastics in the water samples were 0.5–3.1 n L^−1^, while the abundances of microplastics in the sediments were 15–160 n kg^−1^dw^−1^ (the amount of microplastics contained in each kilogram of dry sediment sample), both of which were lower than the abundances of microplastics in the water bodies of the studied parks (27). Similarly, The sediment samples from seven locations at different river levels in Shanghai were collected for microplastics extraction and separation and reported that the abundances of microplastics in the sediments ranged from 53 to 1,600 n kg^−1^ dw^−1^ (58). A possible reason was that the total water volume and watershed area of the parks in Shanghai were much lower than those of the parks in this study, and the dilution and purification ability for treating land point and non-point source pollution from the same area was weak, resulting in a high concentration of microplastics in small water bodies in the area. The aperture size of the sampling tool also affected the abundances and types of microplastics detected (11). There are still no established standard field protocols or sampling methods for microplastics worldwide. Previous studies used different sample collection methods and quantification units, making it difficult to compare data from different studies. Most microplastics research, especially in marine environments, uses trawls with aperture sizes greater than 300 μm (35). Compared with those in the other surveyed waters, the concentration of microplastics in the water of each park was significantly greater. This partly explains the severe microplastic pollution detected in the park. On the other hand, the small-aperture sampler used in this study increased the collection of certain forms of microplastics, such as fibers, by several orders of magnitude compared to that of the larger aperture trawl, which may also be one of the reasons for the significantly greater abundances of microplastics in this study.

In this study, there were differences in the shapes and polymer types of the microplastics in surface waters and sediments. The water microplastics included PET, CPE, and FLU, while the sediment microplastics comprised PVC, ACR, and CP. PET is commonly used to make clothing and water bottles (59). This fibrous polymer was widely detected among the microplastics, and it was usually attributable to the release of synthetic fibers from textiles and clothing during washing. During the laundry process, every 6 kg of clothing could release 1,900–700,000 fibers into the environment (60). PVC was once the world’s largest component among general-purpose plastics and is widely used as a raw material for thin-film plastics (61). It is widely used in building materials, industrial products, daily necessities, floor leather, floor tiles, artificial leather, pipes, wires and cables, packaging films, bottles, foaming materials, sealing materials, fibers, and other fields (62). The main source of PVC in the sediments was likely the degradation of fishing nets or fishing lines (63). Interestingly, although the densities of polyester (1.37 g cm^−3^), nylon (1.15 g cm^−3^), and polystyrene (1.05 g cm^−3^) are greater than those of water, these plastics were still present on the surface of the water. This indicated that in addition to density, other factors could also affect the vertical distributions of microplastics in water. First, the large specific surface areas of microplastics allow these plastic particles to be suspended in water (53). Second, physical factors, such as the temperature, pressure, and wind disturbances, could resuspend underwater microplastics to the surface of the water. Furthermore, biological attachment may also alter the overall densities of plastic fragments, causing them to naturally sink or float (64). Therefore, the vertical distributions of microplastics in water are determined by their own properties and external environmental factors. Different polymers have different densities, and their densities and shapes together determine the states of microplastics in water environments, whether floating, suspended or settling. However, from a methodological perspective, this study used a saturated NaCl solution for flotation of the microplastics in sediment. Therefore, the inconsistency in the results may be related to the hydrological and physicochemical characteristics of different water bodies. On the one hand, microplastics in the water environment may flip or migrate with water flow; on the other hand, the physical and chemical properties of the water environment, including the pH, water temperature, and biomass, affect the status of the microplastics. Microplastics in the water may settle into the sediment in the form of feces after being ingested by organisms or may be covered by algae or bacteria with increased density and settle into the sediment (65). However, further research is needed.

Studies have shown that there are positive correlations between microplastics pollution and human activities; with more frequent human activities, the microplastics pollution problem is more serious (10, 66). There are 120,000 residents around MX, 90,000 people around YD and 70,000 people around YHD, and these parks have the highest microplastics contents and risk levels. The amounts of microplastics in water are influenced by many factors, such as the population density around the water, the distance from the city center, the distance from the mainstream of the river, the precipitation level, water retention time, water surface area, waste management mode, sewage overflow and air sedimentation (67, 68). Many of these factors are closely related to the population density around the park. The lower reaches of the Yangtze River in China have larger surrounding populations and higher plastics production and usage levels than the middle and upper reaches of the Yangtze River basin. Frequent human activities may lead to severe microplastics pollution in small water bodies (30). Regression analyses also indicated that the population and industrial and domestic wastewater discharge had the strongest correlations with the abundance of microplastics in park water. Currently, the vast majority of sewage treatment plants do not have the ability to remove microplastics, so wastewater discharge from sewage treatment plants is also an important source of microplastics in water environments (69, 70). In many countries and regions, microplastics are not included within the monitoring range of wastewater pollutants from sewage treatment plants, and most sewage treatment plants lack advanced devices (such as tertiary treatment) with which to remove microplastics from the wastewater (12, 65). Surprisingly, the regression analyses in this study showed that the correlations of car flow and tourists with the abundances of microplastics in the park water bodies were the weakest. Numerous studies have shown that microplastics generated by automobile tire wear enter aquatic environments through road or rainwater runoff, and they are the largest proportion of microplastics in water bodies (71, 72). There was also a strong correlation between tourism income and the abundance of microplastics. Forty-four percent of the trash generated by tourists is plastic waste (73), and the sources of trash in scenic areas are scattered and difficult to control. In addition, a serious shortage of trash bins during peak tourist seasons and inadequate cleaning by staff lead to poor management of plastic waste and easy entry into the environment, including microplastics pollution of water. The reason for this may be that the overall environmental appearance and tourist attractions of Changsha city are relatively high, and there is limited littering at will. At the same time, sanitation workers also cleaned in a timely manner.

## 5 Conclusion

This article studied the spatial distributions and compositions of microplastics in eight typical parks in the city. The results showed that there were a large amount of microplastics (120–585 n kg^−1^) in the water and sediment of these parks, which posed a certain safety risk. In parks located in densely populated areas, the abundance and PERIs of microplastics were also the highest, indicating that people’s daily activities were the main cause of microplastic pollution in parks. On the contrary, the correlation between car flow and tourists and the abundance of microplastics in park water and sediment was the weakest, indicating that the management of urban parks was very standardized and tourists’ behaviors were also very standardized. Further analysis showed that the microplastics in urban park water were mainly PET, CPE, and FLU, while those in sediment were mainly PVC, ACR, and CPE. These microplastics mainly originated from domestic sewage in residential areas and industrial emissions. Therefore, we recommend that government personnel and urban park managers should pay more attention to the harm caused by residential areas around the park to park, strengthen corresponding management and governance, and should not focus too much on the flow of car flow and tourists on urban roads.

## Data availability statement

The raw data supporting the conclusions of this article will be made available by the authors, without undue reservation.

## Author contributions

JY: Conceptualization, Data curation, Formal analysis, Methodology, Writing – original draft, Writing – review & editing, Investigation. JL: Writing – review & editing, Investigation. JQ: Investigation, Writing – review & editing. MW: Investigation, Writing – review & editing. LT: Investigation, Writing – review & editing. HH: Investigation, Writing – review & editing, Formal analysis, Methodology. KT: Investigation, Writing – review & editing, Methodology, Visualization. SL: Writing – review & editing, Conceptualization, Data curation, Funding acquisition, Methodology, Project administration, Resources, Supervision.

## Footnotes

^1^https://www.r-project.org

## References

[1] Fan C Huang Y-Z Lin J-N Li J. Microplastic constituent identification from admixtures by Fourier-transform infrared (FTIR) spectroscopy: the use of polyethylene terephthalate (PET), polyethylene (PE), polypropylene (PP), polyvinyl chloride (PVC) and nylon (NY) as the model constituents. Environ Technol Innovat. (2021) 23:101798. doi: 10.1016/j.eti.2021.101798

[2] Yang J Xu Y-P Chen P Li J-Y Liu D Chu X-L. Combining spectroscopy and machine learning for rapid identification of plastic waste: recent developments and future prospects. J Clean Prod. (2023) 431:139771. doi: 10.1016/j.jclepro.2023.139771

[3] Nyaga MP Shabaka S Oh S Osman DM Yuan W Zhang W. Microplastics in aquatic ecosystems of Africa: a comprehensive review and meta-analysis. Environ Res. (2024) 248:118307. doi: 10.1016/j.envres.2024.118307 38307187

[4] Yang S Zhou M Chen X Hu L Xu Y Fu W. A comparative review of microplastics in lake systems from different countries and regions. Chemosphere. (2022) 286:131806. doi: 10.1016/j.chemosphere.2021.131806 34426137

[5] Bhutto SUA Akram M You X-y. Probabilistic risk assessment of microplastics in tai Lake, China. Sci Total Environ. (2024) 914:169965. doi: 10.1016/j.scitotenv.2024.169965 38211859

[6] Anagha PL Viji NV Devika D Ramasamy EV. Distribution and abundance of microplastics in the water column of Vembanad Lake–a Ramsar site in Kerala, India. Mar Pollut Bull. (2023) 194:115433. doi: 10.1016/j.marpolbul.2023.115433 37643529

[7] Jung YS Sampath V Prunicki M Aguilera J Allen H LaBeaud D. Characterization and regulation of microplastic pollution for protecting planetary and human health. Environ Pollut. (2022) 315:120442. doi: 10.1016/j.envpol.2022.120442 36272609 PMC12077371

[8] Mamun AA Prasetya TAE Dewi IR Ahmad M. Microplastics in human food chains: food becoming a threat to health safety. Sci Total Environ. (2023) 858:159834. doi: 10.1016/j.scitotenv.2022.159834 36461575

[9] Yang Y Chen L Xue L. Looking for a Chinese solution to global problems: the situation and countermeasures of marine plastic waste and microplastics pollution governance system in China. Chin J Popul Resour Environ. (2021) 19:352–7. doi: 10.1016/j.cjpre.2022.01.008

[10] Jain R Gaur A Suravajhala R Chauhan U Pant M Tripathi V. Microplastic pollution: understanding microbial degradation and strategies for pollutant reduction. Sci Total Environ. (2023) 905:167098. doi: 10.1016/j.scitotenv.2023.167098 37717754

[11] Rafiq A Xu J-L. Microplastics in waste management systems: a review of analytical methods, challenges and prospects. Waste Manag. (2023) 171:54–70. doi: 10.1016/j.wasman.2023.08.015 37647726

[12] Fu Z Wang J. Current practices and future perspectives of microplastic pollution in freshwater ecosystems in China. Sci Total Environ. (2019) 691:697–712. doi: 10.1016/j.scitotenv.2019.07.167 31325868

[13] Zeb A Liu W Ali N Shi R Wang Q Wang J. Microplastic pollution in terrestrial ecosystems: global implications and sustainable solutions. J Hazard Mater. (2024) 461:132636. doi: 10.1016/j.jhazmat.2023.132636 37778309

[14] Liu R-p Li Z-z Liu F Dong Y Jiao J-g Sun P-p. Microplastic pollution in Yellow River, China: current status and research progress of biotoxicological effects. China Geol. (2021) 4:585–92. doi: 10.31035/cg2021081

[15] Guo S Zhang J Liu J Guo N Zhang L Wang S. Organic fertilizer and irrigation water are the primary sources of microplastics in the facility soil, Beijing. Sci Total Environ. (2023) 895:165005. doi: 10.1016/j.scitotenv.2023.165005 37353032

[16] Thacharodi A Meenatchi R Hassan S Hussain N Bhat MA Arockiaraj J. Microplastics in the environment: a critical overview on its fate, toxicity, implications, management, and bioremediation strategies. J Environ Manag. (2024) 349:119433. doi: 10.1016/j.jenvman.2023.119433 39492398

[17] Alimi OS Claveau-Mallet D Lapointe M Biu T Liu L Hernandez LM. Effects of weathering on the properties and fate of secondary microplastics from a polystyrene single-use cup. J Hazard Mater. (2023) 459:131855. doi: 10.1016/j.jhazmat.2023.131855 37478596

[18] Guo C Wang L Lang D Qian Q Wang W Wu R. UV and chemical aging alter the adsorption behavior of microplastics for tetracycline. Environ Pollut. (2023) 318:120859. doi: 10.1016/j.envpol.2022.120859 36521717

[19] Hu K Tian W Yang Y Nie G Zhou P Wang Y. Microplastics remediation in aqueous systems: strategies and technologies. Water Res. (2021) 198:117144. doi: 10.1016/j.watres.2021.117144 33933920

[20] Pastorino P Anselmi S Zanoli A Esposito G Bondavalli F Dondo A. The invasive red swamp crayfish (*Procambarus clarkii*) as a bioindicator of microplastic pollution: insights from Lake Candia (northwestern Italy). Ecol Indic. (2023) 150:110200. doi: 10.1016/j.ecolind.2023.110200

[21] Zhao M Cao Y Chen T Li H Tong Y Fan W. Characteristics and source-pathway of microplastics in freshwater system of China: a review. Chemosphere. (2022) 297:134192. doi: 10.1016/j.chemosphere.2022.134192 35257703

[22] Laju RL Jayanthi M Jeyasanta KI Patterson J Bilgi DS Sathish N. Microplastic contamination in Indian rural and urban lacustrine ecosystems. Sci Total Environ. (2023) 895:165146. doi: 10.1016/j.scitotenv.2023.165146 37385488

[23] Kunz A Schneider F Anthony N Lin H-T. Microplastics in rivers along an urban-rural gradient in an urban agglomeration: correlation with land use, potential sources and pathways. Environ Pollut. (2023) 321:121096. doi: 10.1016/j.envpol.2023.121096 36657513

[24] Faure F Demars C Wieser O Kunz M de Alencastro LF. Plastic pollution in Swiss surface waters: nature and concentrations, interaction with pollutants. Environ Chem. (2015) 12:582–91. doi: 10.1071/EN14218

[25] Eriksen M Mason S Wilson S Box C Zellers A Edwards W. Microplastic pollution in the surface waters of the Laurentian Great Lakes. Mar Pollut Bull. (2013) 77:177–82. doi: 10.1016/j.marpolbul.2013.10.007 24449922

[26] Fischer EK Paglialonga L Czech E Tamminga M. Microplastic pollution in lakes and Lake shoreline sediments – a case study on Lake Bolsena and Lake Chiusi (Central Italy). Environ Pollut. (2016) 213:648–57. doi: 10.1016/j.envpol.2016.03.012 27104923

[27] Su L Cai H Kolandhasamy P Wu C Rochman CM Shi H. Using the Asian clam as an indicator of microplastic pollution in freshwater ecosystems. Environ Pollut. (2018) 234:347–55. doi: 10.1016/j.envpol.2017.11.075 29195176

[28] Wang W Ndungu AW Li Z Wang J. Microplastics pollution in inland freshwaters of China: a case study in urban surface waters of Wuhan, China. Sci Total Environ. (2017) 575:1369–74. doi: 10.1016/j.scitotenv.2016.09.213 27693147

[29] Luo W Su L Craig NJ Du F Wu C Shi H. Comparison of microplastic pollution in different water bodies from urban creeks to coastal waters. Environ Pollut. (2019) 246:174–82. doi: 10.1016/j.envpol.2018.11.081 30543943

[30] Zhang K Shi H Peng J Wang Y Xiong X Wu C. Microplastic pollution in China's inland water systems: a review of findings, methods, characteristics, effects, and management. Sci Total Environ. (2018) 630:1641–53. doi: 10.1016/j.scitotenv.2018.02.300 29554780

[31] Nosova AO Uspenskaya MV. Ecotoxicological effects and detection features of polyvinyl chloride microplastics in soils: a review. Environ Adv. (2023) 13:100437. doi: 10.1016/j.envadv.2023.100437

[32] Xue Q Wang N Yang H Yang J Bai H. Detection of microplastics based on spatial heterodyne Raman spectroscopy. Spectrochim Acta A Mol Biomol Spectrosc. (2022) 283:121712. doi: 10.1016/j.saa.2022.121712 35952588

[33] González-Pleiter M Edo C Velázquez D Casero-Chamorro MC Leganés F Quesada A. First detection of microplastics in the freshwater of an Antarctic specially protected area. Mar Pollut Bull. (2020) 161:111811. doi: 10.1016/j.marpolbul.2020.111811 33157507

[34] Dong H Wang X Niu X Zeng J Zhou Y Suona Z. Overview of analytical methods for the determination of microplastics: current status and trends. TrAC Trends Anal Chem. (2023) 167:117261. doi: 10.1016/j.trac.2023.117261

[35] Nousheen R Hashmi I Rittschof D Capper A. Comprehensive analysis of spatial distribution of microplastics in Rawal Lake, Pakistan using trawl net and sieve sampling methods. Chemosphere. (2022) 308:136111. doi: 10.1016/j.chemosphere.2022.136111 35995190

[36] Lv W Zhou W Lu S Huang W Yuan Q Tian M. Microplastic pollution in rice-fish co-culture system: a report of three farmland stations in Shanghai, China. Sci Total Environ. (2019) 652:1209–18. doi: 10.1016/j.scitotenv.2018.10.321 30586807

[37] Zhou Q Chen J Zhang D Pan X. Evaluation of organic matter removal by H2O2 from microplastic surface by nano-physicochemical methods. Green Anal Chem. (2022) 3:100035. doi: 10.1016/j.greeac.2022.100035

[38] la Cecilia D Philipp M Kaegi R Schirmer M Moeck C. Microplastics attenuation from surface water to drinking water: impact of treatment and managed aquifer recharge – and identification uncertainties. Sci Total Environ. (2024) 908:168378. doi: 10.1016/j.scitotenv.2023.168378 37951258

[39] Nan B Su L Kellar C Craig NJ Keough MJ Pettigrove V. Identification of microplastics in surface water and Australian freshwater shrimp *Paratya australiensis* in Victoria. Aus Environ Pollut. (2020) 259:113865. doi: 10.1016/j.envpol.2019.113865 31891912

[40] Li Y Fu J Peng L Sun X Wang G Wang Y. A sustainable emulsion for separation and Raman identification of microplastics and nanoplastics. Chem Eng J. (2023) 469:143992. doi: 10.1016/j.cej.2023.143992

[41] Huang L Li QP Li H Yuan X. Microplastic pollution and regulating factors in the surface sediment of the Xuande atolls in the South China Sea. Mar Pollut Bull. (2023) 196:115562. doi: 10.1016/j.marpolbul.2023.115562 37769406

[42] Everaert G Van Cauwenberghe L De Rijcke M Koelmans AA Mees J Vandegehuchte M. Risk assessment of microplastics in the ocean: modelling approach and first conclusions. Environ Pollut. (2018) 242:1930–8. doi: 10.1016/j.envpol.2018.07.069 30061084

[43] Lithner D Larsson Å Dave G. Environmental and health hazard ranking and assessment of plastic polymers based on chemical composition. Sci Total Environ. (2011) 409:3309–24. doi: 10.1016/j.scitotenv.2011.04.038 21663944

[44] Montecinos S Tognana S Salgueiro W Frosinini C. Temporal variation of the microplastic concentration in a stream that receives discharge from wastewater treatment plants. Environ Pollut. (2024) 340:122776. doi: 10.1016/j.envpol.2023.122776 37871739

[45] Yu Y Zhang Z Zhang Y Jia H Li Y Yao H. Abundances of agricultural microplastics and their contribution to the soil organic carbon pool in plastic film mulching fields of Xinjiang, China. Chemosphere. (2023) 316:137837. doi: 10.1016/j.chemosphere.2023.137837 36640972

[46] Burghardt TE Pashkevich A. Road markings and microplastics – a critical literature review. Transp Res Part D: Transp Environ. (2023) 119:103740. doi: 10.1016/j.trd.2023.103740

[47] Ponti MG Allen D White CJ Bertram D Switzer C. A framework to assess the impact of flooding on the release of microplastics from waste management facilities. J Hazard Mater Adv. (2022) 7:100105. doi: 10.1016/j.hazadv.2022.100105

[48] Bozkurt HS Yörüklü HC Bozkurt K Denktaş C Bozdoğan A Özdemir O. Biodegradation of microplastic by probiotic bifidobacterium. Int J Global Warming. (2022) 26:429–43. doi: 10.1504/IJGW.2022.122435

[49] Giangeri G Morlino MS De Bernardini N Ji M Bosaro M Pirillo V. Preliminary investigation of microorganisms potentially involved in microplastics degradation using an integrated metagenomic and biochemical approach. Sci Total Environ. (2022) 843:157017. doi: 10.1016/j.scitotenv.2022.157017 35777567

[50] Kim J Maruthupandy M An KS Lee KH Jeon S Kim J-S. Acute and subacute repeated oral toxicity study of fragmented microplastics in Sprague-Dawley rats. Ecotoxicol Environ Saf. (2021) 228:112964. doi: 10.1016/j.ecoenv.2021.112964 34773844

[51] Zhao W Li J Liu M Wang R Zhang B Meng X-Z. Seasonal variations of microplastics in surface water and sediment in an inland river drinking water source in southern China. Sci Total Environ. (2024) 908:168241. doi: 10.1016/j.scitotenv.2023.168241 37914114

[52] Cera A Pierdomenico M Sodo A Scalici M. Spatial distribution of microplastics in volcanic lake water and sediments: relationships with depth and sediment grain size. Sci Total Environ. (2022) 829:154659. doi: 10.1016/j.scitotenv.2022.154659 35307421

[53] Zhao S Zhu L Li D. Microplastic in three urban estuaries, China. Environ Pollut. (2015) 206:597–604. doi: 10.1016/j.envpol.2015.08.027 26312741

[54] Peller J Nevers MB Byappanahalli M Nelson C Ganesh Babu B Evans MA. Sequestration of microfibers and other microplastics by green algae, Cladophora, in the US Great Lakes. Environ Pollut. (2021) 276:116695. doi: 10.1016/j.envpol.2021.116695 33601201

[55] Zhang L Liu J Xie Y Zhong S Gao P. Occurrence and removal of microplastics from wastewater treatment plants in a typical tourist city in China. J Clean Prod. (2021) 291:125968. doi: 10.1016/j.jclepro.2021.125968

[56] Castillo C Fernández C Gutiérrez MH Aranda M Urbina MA Yáñez J. Water column circulation drives microplastic distribution in the Martínez-baker channels; a large fjord ecosystem in Chilean Patagonia. Mar Pollut Bull. (2020) 160:111591. doi: 10.1016/j.marpolbul.2020.111591 32898738

[57] Napper IE Baroth A Barrett AC Bhola S Chowdhury GW Davies BFR. The distribution and characterisation of microplastics in air, surface water and sediment within a major river system. Sci Total Environ. (2023) 901:166640. doi: 10.1016/j.scitotenv.2023.166640 37647965

[58] Peng G Xu P Zhu B Bai M Li D. Microplastics in freshwater river sediments in Shanghai, China: a case study of risk assessment in mega-cities. Environ Pollut. (2018) 234:448–56. doi: 10.1016/j.envpol.2017.11.034 29207296

[59] Bai B Liu Y Zhang H Zhou F Han X Wang Q. Experimental investigation on gasification characteristics of polyethylene terephthalate (PET) microplastics in supercritical water. Fuel. (2020) 262:116630. doi: 10.1016/j.fuel.2019.116630

[60] Scopetani C Esterhuizen-Londt M Chelazzi D Cincinelli A Setälä H Pflugmacher S. Self-contamination from clothing in microplastics research. Ecotoxicol Environ Saf. (2020) 189:110036. doi: 10.1016/j.ecoenv.2019.110036 31825795

[61] Peng B-Y Sun Y Zhang X Sun J Xu Y Xiao S. Unveiling the residual plastics and produced toxicity during biodegradation of polyethylene (PE), polystyrene (PS), and polyvinyl chloride (PVC) microplastics by mealworms (larvae of *Tenebrio molitor*). J Hazard Mater. (2023) 452:131326. doi: 10.1016/j.jhazmat.2023.131326 37027925

[62] Gulizia AM Philippa B Zacharuk J Motti CA Vamvounis G. Plasticiser leaching from polyvinyl chloride microplastics and the implications for environmental risk assessment. Mar Pollut Bull. (2023) 195:115392. doi: 10.1016/j.marpolbul.2023.115392 37690404

[63] Cole M Lindeque P Halsband C Galloway TS. Microplastics as contaminants in the marine environment: a review. Mar Pollut Bull. (2011) 62:2588–97. doi: 10.1016/j.marpolbul.2011.09.025 22001295

[64] Li J Liu H Paul CJ. Microplastics in freshwater systems: a review on occurrence, environmental effects, and methods for microplastics detection. Water Res. (2018) 137:362–74. doi: 10.1016/j.watres.2017.12.056 29580559

[65] Li Y Wu M Li H Xue H Tao J Li M. Current advances in microplastic contamination in aquatic sediment: analytical methods, global occurrence, and effects on elemental cycling. TrAC Trends Anal Chem. (2023) 168:117331. doi: 10.1016/j.trac.2023.117331

[66] Wang Y Zhong Z Chen X Sokolova I Ma L Yang Q. Microplastic pollution and ecological risk assessment of Yueqing Bay affected by intensive human activities. J Hazard Mater. (2024) 461:132603. doi: 10.1016/j.jhazmat.2023.132603 37778312

[67] Ismanto A Hadibarata T Sugianto DN Zainuri M Kristanti RA Wisha UJ. First evidence of microplastics in the water and sediment of Surakarta city river basin, Indonesia. Marine Pollut Bull. (2023) 196:115677. doi: 10.1016/j.marpolbul.2023.115677 37862842

[68] Koelmans AA Mohamed Nor NH Hermsen E Kooi M Mintenig SM De France J. Microplastics in freshwaters and drinking water: critical review and assessment of data quality. Water Res. (2019) 155:410–22. doi: 10.1016/j.watres.2019.02.054 30861380 PMC6449537

[69] Lessa Belone MC Brosens D Kokko M Sarlin E. Effects of mesophilic and thermophilic anaerobic digestion of sewage sludge on different polymers: perspectives on the potential of the treatment to degrade microplastics. Sci Total Environ. (2024) 907:168014. doi: 10.1016/j.scitotenv.2023.168014 37871819

[70] Hong Y Oh J Lee I Fan C Pan S-Y Jang M. Total-organic-carbon-based quantitative estimation of microplastics in sewage. Chem Eng J. (2021) 423:130182. doi: 10.1016/j.cej.2021.130182

[71] Liu Y Chen H Wu S Gao J Li Y An Z. Impact of vehicle type, tyre feature and driving behaviour on tyre wear under real-world driving conditions. Sci Total Environ. (2022) 842:156950. doi: 10.1016/j.scitotenv.2022.156950 35753475

[72] Luo Z Zhou X Su Y Wang H Yu R Zhou S. Environmental occurrence, fate, impact, and potential solution of tire microplastics: similarities and differences with tire wear particles. Sci Total Environ. (2021) 795:148902. doi: 10.1016/j.scitotenv.2021.148902 34328941

[73] Truchet DM López ADF Ardusso MG Rimondino GN Buzzi NS Malanca FE. Microplastics in bivalves, water and sediments from a touristic sandy beach of Argentina. Mar Pollut Bull. (2021) 173:113023. doi: 10.1016/j.marpolbul.2021.113023 34695691

